# Radiation pneumonitis after concurrent aumolertinib and thoracic radiotherapy in EGFR-mutant non-small cell lung cancer patients

**DOI:** 10.1186/s12885-024-11946-y

**Published:** 2024-02-12

**Authors:** Hanjing Yin, Wenxiao Jia, Jinming Yu, Hui Zhu

**Affiliations:** 1 Department of Radiation Oncology, Shandong Cancer Hospital and Institute, Shandong First Medical University and Shandong Academy of Medical Sciences, Jinan, 250117 China; 2 Cancer Center, Union Hospital, Tongji Medical College, Huazhong University of Science and Technology, 109 Machang Road, Wuhan, 430022 Hubei China; 3 Department of Radiation Oncology, Shandong Cancer Hospital and Institute, Shandong First Medical University and Shandong Academy of Medical Sciences, 440 Jiyan Road, Jinan, 250117 Shandong China

**Keywords:** Radiation pneumonitis, EGFR-TKI, Aumolertinib, Thoracic radiotherapy, Non-small cell lung cancer

## Abstract

**Background:**

The superior efficacy of concurrent thoracic radiotherapy (TRT) and epidermal growth factor receptor tyrosine kinase inhibitors (EGFR-TKIs) has been proven in locally advanced and advanced non-small cell lung cancer (NSCLC) patients with EGFR mutations. However, the high incidence of radiation pneumonitis (RP) reduced by concurrent TRT and TKIs has attracted widespread attention. Thus, this study was designed to investigate the rate and risk factors for RP in EGFR-positive NSCLC patients simultaneously treated with aumolertinib and TRT.

**Methods:**

We retrospectively evaluated stage IIIA-IVB NSCLC patients treated with concurrent aumolertinib and TRT between May 2020 and December 2022 at Shandong Cancer Hospital and Institute, Shandong, China. RP was diagnosed by two senior radiologists and then graded from 1 to 5 according to the Common Terminology Criteria for Adverse Events v5.0. All risk factors were evaluated by univariate and multivariate logistic regression analyses.

**Results:**

A total of 49 patients were included, the incidence of grade ≥ 2 RP was 42.9%. Grade 2 and 3 RP were observed in 28.6% and 14.3% of patients, respectively. Grade 4 to 5 RP were not observed. the gross total volume (GTV) ≥ 21 ml and ipsilateral lung V20 ≥ 25% were risk factors for RP. The median progression-free survival (PFS) in the first-line therapy group and second-line therapy group were 23.5 months and 17.2 months, respectively (*p* = 0.10).

**Conclusions:**

Better local control is achieved with concurrent TRT and aumolertinib, and special attention should be given to controlling ipsilateral lung V20 and GTV to reduce the risk of RP.

## Background

Epidermal growth factor receptor tyrosine kinase inhibitors (EGFR-TKIs) are regarded as the standard treatment for stage IV EGFR-mutant non-small cell lung cancer (NSCLC) patients [1], but 50–70% of patients have developed T790M mutations associated with resistance after receiving the first- or second-generation TKIs [2, 3]. Subsequently, AURA studies demonstrated that the survival advantages offered by osimertinib surpassed those of first-generation TKIs for EGFR-TKI-resistant patients [4–6]. The FLAURA study further demonstrated the superior clinical efficacy of osimertinib as a first-line therapy in EGFR-mutant NSCLC patients, and the median progression-free survival (PFS) was 18.9 months [7]. Subsequently, the AENEAS study proved that the use of aumolertinib as the first-line therapy further prolonged PFS to 19.3 months, and safety was well tolerated [8]. Overall, osimertinib and aumolertinib are regarded as the first-line standard therapies for EGFR-mutant NSCLC patients.

Notably, most patients inevitably experienced progressive disease (PD) after receiving 8–18 months of TKI alone [5, 9], and primary lung lesions were the most common sites of initial failure in patients with advanced NSCLC [10, 11]. Therefore, more attention should be given to the local control during the application of TKI. Xu et al. first demonstrated that consolidative local ablative therapy (LAT) combined with TKI improved the median progression-free survival (PFS) (20.6 months versus 15.6 months, *P* < 0.001) of patients with oligometastatic EGFR-mutant NSCLC [12]. The REFRACT study, the largest analysis of Chinese patients, provided strong evidence for the first time that thoracic radiotherapy (TRT) combined with TKI was the preferred treatment option compared to TKI or radiation alone [13]. Several other studies reached a similar conclusion, the addition of radiotherapy was beneficial for local control and prolonged PFS [14–16]. Notably, Zhou et al. and Wei et al. reported that preemptive addition of TRT before PD provided superior PFS and favorable safety in patients treated with 3 months of TKI [17, 18]. One study revealed that tumors usually shrink quickly within 40 days after TKI, and it was difficult for tumors to shrink after 90 days [19]. Therefore, early radiation therapy is indeed beneficial for survival, although the optimal timing for early radiation therapy has not yet been determined.

Due to the importance of combined TRT, the occurrence of radiation pneumonitis (RP) cannot be ignored. Zheng et al. explored the incidence of RP in NSCLC patients who received first-generation TKIs combined with concurrent TRT and reported that 30% of patients experienced grade ≥ 2 RP, and grade 5 RP occurred in 10% of patients [20]. With respect to the combination of osimertinib and TRT, our team previously reported that 63.6% of patients experienced grade ≥ 2 RP, 5 patients (45.4%) exhibited grade 3 RP, and 1 (9.1%) patient died [21], suggesting that additional caution should be taken in the application of third-generation TKI.

Given that NSCLC patients with EGFR activating mutations has accounted for 50% of Asian patients [22], aumolertinib as a third-generation TKI made in China brought more options for NSCLC patients especially in the Asian region. However, the exact clinical efficacy and rate of RP among NSCLC patients treated with the combination of aumolertinib and TRT have not been completely evaluated. Therefore, in this study, we first explore the clinical efficacy and incidence of RP in Chinese patients treated with concurrent TRT and aumolertinib, and further analyses the risk factors for RP.

## Patients and methods

### Patients

We retrospectively collected the data of NSCLC patients treated with TRT and aumolertinib at Shandong Cancer Hospital and Institute between May 2020 and December 2022. The inclusion criteria were as follows: (1) stage IIIA-IVB EGFR-mutant NSCLC, and (2)received concurrent TRT and aumolertinib. Patients with a history of TRT or interstitial lung disease or those treated with immune checkpoint inhibitors were excluded. This study included a total of 8 patients with stage III NSCLC, 2 patients with stage IIIA NSCLC and the remaining patients with stage IIIB NSCLC. One of the patients did not meet the indications for surgery due to poor cardiac function and a history of cerebral infarction. The patient and their families refused chemotherapy and therefore received TRT combined with aumolertinib. Another patient was too old to tolerate surgery, and he also refused chemoradiotherapy after discussion with his family. Simultaneous treatment was defined as at least one day overlap existed between aumolertinib and TRT. The overlap time was the number of days when TRT and aumolertinib were administered simultaneously. This study was approved by the Institutional Review Board of Shandong Cancer Hospital and Institute and was performed in accordance with the Declaration of Helsinki.

### Treatment protocol

All patients were treated with aumolertinib as the first-line or second-line therapy. Aumolertinib was taken orally at 110 mg/d until uncontrollable PD or intolerable toxicity occurred. The primary endpoint of this study was the occurrence of RP, and the second primary endpoint was PFS. During the application of TKI, patients who had stable disease (SD) were treated with concurrent TRT as consolidative therapy, while patients who experienced PD were treated with TRT to control disease progression. Before undergoing intensity-modulated radiotherapy (IMRT), all patients underwent computed tomography (CT) stimulation via a Philips 16-slice Brilliance big-bore computed tomography scanner (Philips Medical Systems, Amsterdam, Netherlands) with a 3 mm slice thickness, and the CT images were imported into an Eclipse 15.5 (Varian, USA) planning system for target and organ-at-risk (OAR) contouring. The gross tumor volume (GTV) was defined as the primary tumor and lymph node metastasis visible on CT, and the planning target volume (PTV) was defined as the GTV with a 6–8 mm uniform expansion. Dosimetric parameters were extracted from a dose and volume histogram (DVH) in the Eclipse 15.5 system. The lung Vdose (from V5 to V30) was defined as the percentage of total lung volume or the affected lung volume receiving equal to or greater than the designated dose of radiation. In some patients, the second treatment plan was repeated, and all CT images and RT plans were imported into the MIM Maestro (version 7.1.7, USA) system for deformable registration and dose superimposition to obtain new dosimetric parameters.

### Diagnosis and classification of radiation pneumonitis

RP was diagnosed by two senior radiologists based on patients’ symptom, laboratory test results and CT images. RP was graded from 1 to 5 according to the Common Terminology Criteria for Adverse Events v5.0 [23]. Grade 1 RP was asymptomatic pneumonitis or minimal symptoms that did not require intervention; grade 2 RP was accompanied by coughing, chest distress or other symptoms that did not interfere with daily activities and needed symptomatic treatment; grade 3 RP exhibited severe symptoms and required corticosteroids or the administration of oxygen; grade 4 RP required urgent intervention, such as ventilation, for life-threatening respiratory symptoms; and grade 5 RP was fatal.

### Statistical analysis

Binary logistic regression analyses were used to explore the correlation between clinical factors and grade ≥ 2 RP. The area under the curve (AUC) of the receiver operating characteristic (ROC) curve was calculated to evaluate the ability of continuous variables to predict RP, and the optimal cutoff value was calculated based on the maximum Youden index to transform continuous variables to categorical variables. All categorical variables were evaluated at the significance level of *P* value < 0.05 by univariate and multivariate logistic regression analyses, and the odds ratio (OR) and 95% confidence interval (CI) were also estimated. The log-rank test was used to evaluate the heterogeneity of the survival analysis. All the statistical analyses were performed using SPSS V27.0 (IBM Corporation, Armonk, NY, USA).

## Results

### Patient characteristics

A total of 49 patients with NSCLC were included in our analysis between May 2020 and December 2022, and the last follow-up time was August 2023. The baseline characteristics are summarized in Table 1. The median age was 62 years (range 26–80 years). A total of 55.1% of the patients were female, and 83.7% had stage IV disease. There were 23 (46.9%) and 26 (53.1%) patients with EGFR exon 19 deletion and exon 21 L858R, respectively, all of whom underwent T790M mutation testing before receiving aumolertinib and TRT as the second-line therapy. The median follow-up time was 16.63 months. Aumolertinib and TRT were given to 26 (53.1%) patients as the first-line therapy and 23 (46.9%) patients as the second-line therapy. The median time from the beginning of aumolertinib to TRT was 68 days, and the median overlap time of TRT and aumolertinib was 32 days.

**Table 1:** Baseline characteristics of included patients

Characteristics	N(%)
Gender(n)	
Male	22 (44.9%)
Female	27 (55.1%)
Age (median, range) (y)	62 (26–80)
Smoking history (n)	
Never	40 (81.6%)
Former or current	9 (19.4%)
Location of the primary site (n)	
Upper lobe	28 (56.6%)
Middle and lower lob	21 (43.4%)
TNM stage(n)	
IIIA-IIIC	8 (16.3%)
IVA	25 (51.0%)
IVB	16 (32.7%)
Type of EGFR mutation(n)	
EGFR exon 19	23 (46.9%)
EGFR exon 21	26 (53.1%)
No. of lines of aumolertinib therapy(n)	
1	26 (53.1%)
2	23 (46.9%)
Time of TKI before TRT (median, range) (d)	68 (0–743)
Total dose(median, range) (Gy)	55 (30–75)
Dose per fraction (median, range) (Gy)	2.2 (1.5–4)
GTV size (median, range) (ml)	24.8 (1.3–236.28)
PTV size (median, range) (ml)	116 (7.5–603.2)
MLD (median, range) (Gy)	6.36 (0.77–13.77)
Progression before TRT (n)	
No	33 (67.3%)
Yes	16 (32.7%)
Overlap time of TKI and TRT (median, range) (d)	32 (12–46)

*EGFR* epidermal growth factor receptor, *GTV* gross tumor volume, *PTV* planning target volume, *TKI* tyrosine kinase inhibitor, *MLD* median lung dose, *TRT* thoracic radiotherapy

### Radiation details

The median total dose and median MLD were 55 Gy (range 30–75 Gy) and 6.36 Gy (range 0.77–13.77 Gy), respectively. All patients were treated with conventional fractional radiotherapy. Except for 33 patients who underwent consolidative TRT, 16 patients accepted TRT after developing PD. In terms of the field of TRT, two patients (4.1%) had only irradiated metastatic lymph nodes, 22 patients (44.9%) had irradiated tumor lesions, and 25 patients (51.0%) had both tumor lesions and lymph nodes irradiated.

### Safety

In total, 45 patients (91.8%) experienced at least one treatment-emergent adverse event (TEAE), and TEAEs ≥ grade 3 accounted for 6.1% (Table 2). The most common TEAEs were blood creatine phosphokinase increased, alanine aminotransferase increased and aspartate aminotransferase increased. There was no dose interruption or death due to TEAEs.

**Table 2:** Treatment-emergent adverse events of any grade and grade ≥ 3

Event	All Grade	Grade ≥ 3
Alanine aminotransferase increased	13 (26.5%)	0
Aspartate aminotransferase increased	11 (22.4%)	0
Blood bilirubin increased	4 (8.2%)	0
Blood creatine phosphokinase increased	14 (28.6%)	3(6.1%)
White blood cell count decreased	9 (18.4%)	0
Neutrophil count decreased	5 (10.2%)	0
Platelet count decreased	15 (30.6%)	0
Rash	10 (20.4%)	0
Pruritus	4 (8.2%)	0
Diarrhea	6 (12.2%)	0

### Incidence of RP

**Fig. 1:**
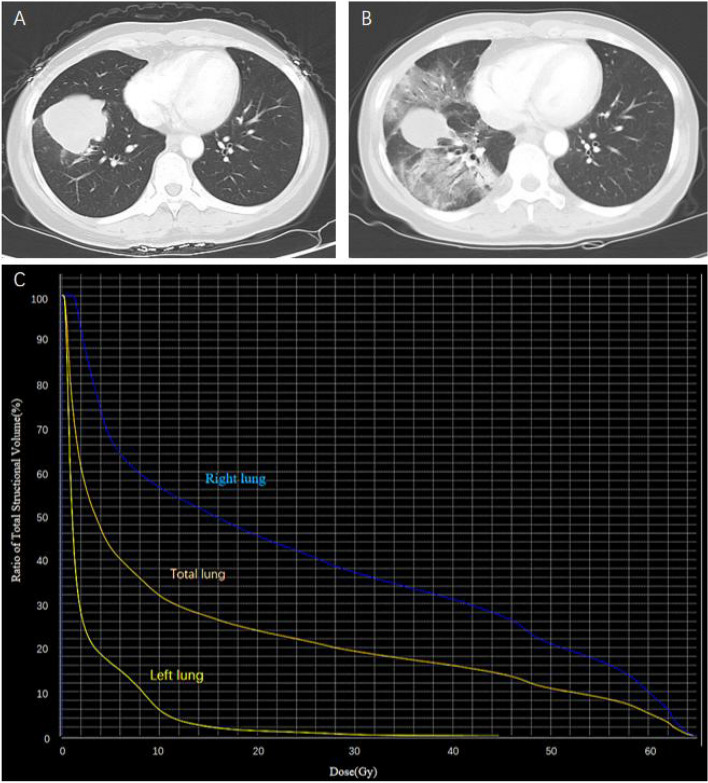
Representative images of one patient who experienced grade 3 RP after concurrent aumolertinib and thoracic radiotherapy. **A** primary lesion before radiotherapy; **B** two months after radiotherapy; **C** dose distribution histogram of the total lung, right lung, and left lung

**Fig. 2:**
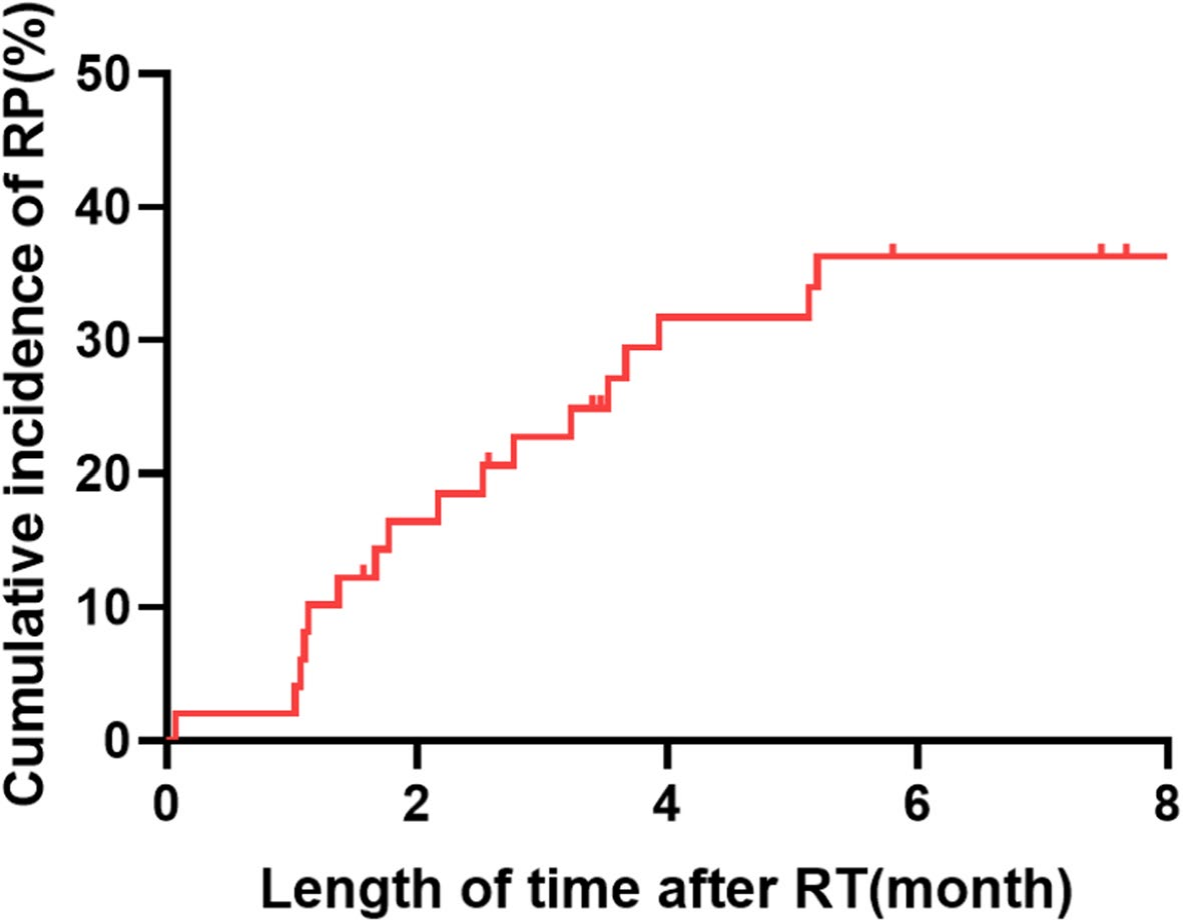
Cumulative incidence curve of grade ≥ 2 RP. RP, radiation pneumonitis; RT radiotherapy

A total of 21 (42.9%) patients experienced grade ≥ 2 RP after TRT combined with aumolertinib. Grade 2 and 3 RP were observed in 28.6% and 14.3% of patients, respectively. Grade 4 to 5 RP were not observed in all patients. Figure 1 showed a typical CT image of a patient with grade 3 RP who was simultaneously treated with TRT and aumolertinib. The patient was a 49-year-old male who received a total of 60 Gy in 20 fractions. The PTV, total mean lung dose (MLD), total lung V20 and ipsilateral lung V20 were 311.07 ml, 13.77 Gy, 23.6% and 44.7%, respectively. The cumulative incidence curve of RP was shown in Fig. 2**.** We found that the incidence of RP rapidly increased one month after the end of TRT and gradually stabilized four months after the end of TRT. The median time from TRT to the occurrence of grade ≥ 2 RP was 2.17 months.

### Risk factors for RP

As shown in Table 3, the MLD, total lung V dose and ipsilateral lung V dose were significantly correlated with grade ≥ 2 RP. In addition, statistically significant correlations were also found between the occurrence of grade ≥ 2 RP and the overlap time of TKI and TRT ≥ 34 days (OR 7.20; 95% CI 1.93–26.81, *P* = 0.003), radiation field of lung tumor plus lymph node (OR 12.73; 95% CI 2.43–66.55, *P* = 0.003), GTV volume ≥ 21 ml (OR 3.24; 95% CI 1.04–10.07, *P* = 0.042), PTV volume ≥ 124 ml (OR 4.28; 95% CI 1.34–13.71, *P* = 0.014) (Table 4).

**Table 3:** Univariate logistic regression analysis of the risk factors for grade ≥ 2 radiation pneumonitis

Factor	*P* value	OR (95% CI)
MLD	**0.001**	1.55 (1.20–1.99)
Total lung V5	**0.006**	1.08 (1.02–1.14)
Total lung V10	**0.004**	1.13 (1.04–1.22)
Total lung V15	**0.002**	1.18 (1.06–1.31)
Total lung V20	**0.001**	1.25 (1.09–1.42)
Total lung V30	**0.001**	1.36 (1.13–1.64)
Ipsilateral lung V5	**0.002**	1.08 (1.03–1.14)
Ipsilateral lung V10	**0.002**	1.10 (1.04–1.17)
Ipsilateral lung V15	**0.002**	1.12 (1.04–1.20)
Ipsilateral lung V20	**0.001**	1.15 (1.06–1.25)
Ipsilateral lung V30	**0.001**	1.20 (1.08–1.34)

*MLD* median lung dose, *OR* odds ratio, *CI* confidence interval

**Table 4:** Univariate logistic regression analysis of the risk factors for radiation pneumonitis

Factor		*P* value	OR (95% CI)
Gender	Male		
	Female	0.327	1.83 (0.55–6.16)
Age	< 58		
	≥ 58	0.091	0.35 (0.10–1.18)
Location of the primary site	Upper lobe		
	Middle and lower lob	0.665	1.30 (0.40–4.25)
TNM stage	IIIA-IIIC	0.580	
	IVA	0.612	0.65 (0.12–3.47)
	IVB	0.770	1.30 (0.23–7.38)
EGFR mutation	EGFR exon 19		
	EGFR exon 21	0.228	0.48 (0.15–1.58)
No. of lines of aumolertinib	1		
	2	0.556	0.70 (0.21–2.29)
Time of TKI before TRT (m)	< 7.7		
	≥ 7.7	0.103	0.26 (0.05–1.32)
Overlap time of TKI and TRT (d)	< 34		
	≥ 34	**0.003**	7.20 (1.93–26.81)
Progression before TRT	No		
	Yes	0.729	0.79 (0.20–3.06)
Total dose (Gy)	< 52		
	≥ 52	0.052	4.12 (0.99–17.16)
Radiation field	Lung tumor alone	**0.010**	
	Lymph node alone	0.149	10.00 (0.44–228.70)
	Lung tumor + lymph node	**0.003**	12.73 (2.43–66.55)
GTV (ml)	< 21		
	≥ 21	**0.001**	35.20 (4.08–303.44)
PTV (ml)	< 116		
	≥ 116	**0.001**	16.50 (3.16–86.26)

*EGFR* epidermal growth factor receptor, *TKI* tyrosine kinase inhibitor, *TRT* thoracic radiotherapy, *GTV* gross tumor volume, *PTV* planning target volume, *OR* odds ratio, *CI* confidence interval

Considering the collinearity of some variables, only the overlap time of TKI and TRT, GTV and ipsilateral lung V20 were included in the multivariate analysis. The results are summarized in Table 5. The GTV and ipsilateral lung V20 were found to be independent predictive factors for grade ≥ 2 RP. Compared with ipsilateral lung V20 < 25%, ipsilateral lung V20 ≥ 25% (OR 7.40; 95% CI 1.21–45.34; *P* = 0.030) increased the risk of pneumonitis by 7.4 times. GTV ≤ 21 ml was also yielded a lower rate of grade ≥ 2 RP than GTV ≥ 21 ml (OR 17.53; 95% CI 1.68–182.92, *P* = 0.017).

**Table 5:** Multivariate logistic regression analyses of the risk factors for radiation pneumonitis

Factor		*P* value	OR (95% CI)
Overlap time of TKI and TRT (d)	< 34		
	≥ 34	0.159	3.68 (0.60–22.52)
GTV volume (ml)	< 21		
	≥ 21	**0.017**	17.53 (1.68–182.92)
Ipsilateral lung V20 (%)	< 25		
	≥ 25	**0.030**	7.40 (1.21–45.34)

*TKI* tyrosine kinase inhibitor, *TRT* thoracic radiotherapy, *GTV* gross tumor volume, *OR* odds ratio, *CI* confidence interval

### Effect of treatment on PFS

**Fig. 3:**
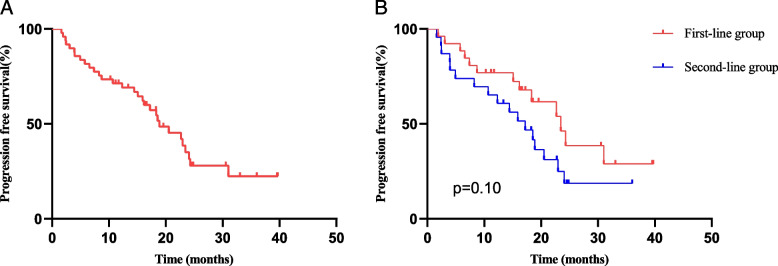
Kaplan‒Meier curves for progression free survival. **A** all patients; **B** patients in first-line group and second-line group

Up to August 2023, 30 patients (61.2%) exhibited disease progression. As showen in Fig. 3, the median PFS of all patients was 18.87 months (95% CI 13.41–24.32). The median PFS in the first-line therapy group and second-line therapy group were 23.5 months and 17.2 months, respectively (*p* = 0.100). Among them, 20 patients experienced progression of chest lesions, 5 patients experienced distant metastasis, and 4 patients presented with both local and distant metastasis simultaneously.

## Discussion

Although the superior clinical efficacy and tolerable safety of aumolertinib have been demonstrated compared with those of first-generation TKI alone,and TRT combined with TKI may be the optimal treatment model, there is also a lack of sufficient experimental data to explore the clinical efficacy and safety of concurrent TRT and aumolertinib. Therefore, our team first explored the incidence and risk factors for RP induced by concurrent TRT and aumolertinib, hoping to provide patients with more efficient and safer treatment plans.

In terms of PFS, the APOLLO study demonstrated that the median PFS was 12.4 months when patients received aumolertinib as the second-line therapy, while AENEAS study demonstrated that the median PFS was 19.3 months when patients received aumolertinib as the first-line therapy [8, 24]. In our study, the median PFS in the first-line therapy group and the second-line therapy group were greater than those in both the AENEAS and APOLLO studies, indicating the superiority of TRT combined with aumolertinib. Although there was no significant difference between the two subgroups (*p* = 0.10), a trend toward benefits from early radiation therapy was still observed. In the REFRACT study, the median PFS of patients treated with RT and first-generation TKI was longer than that in our study (26.2 months versus 23.5 months) [13]. This may be because the patients in the REFRACT study all had locally advanced NSCLC and some patients accepted chemotherapy simultaneously, but the majority of the patients in our study had stage IV disease. In addition, only 61.2% of patients in our study experienced PD, and the overall survival was unclear. Better survival outcomes may be observed as the follow-up time increased. Considering the recency of the launch of aumolertinib in China and the relatively short follow-up time, this study specifically examined the incidence and risk factors of RP. In the future, we will continue to monitor and investigate the impact of radiotherapy and other predictive factors on the survival of EGFR-mutant NSCLC patients treated with aumolertinib.

In terms of RP, numerous studies have explored the occurrence of RP induced by first-generation TKI and TRT. Researchers found that the incidence of grade ≥ 2 RP mostly reached 30%, and more than 10% of patients died from RT [20, 25, 26]. Regarding third-generation TKI, our team previously studied the safety of osimertinib combined with TRT, finding that 63.6% of patients experienced grade ≥ 2 RP and that 9.1% of them experienced grade 5 RP [21]. Although this study involved only 11 people, the extremely high incidence of RP forced us to be cautious about the use of third-generation TKI. To further promote the application of TKI in patients with EGFR mutations in China and even in Asia, it is even more important to study the safety of aumolertinib combined with TRT. Our study revealed that the incidence of RP caused by aumolertinib and TRT was lower than that caused by first-generation TKI, and there were no deaths caused by RP. Although the data from our study were not compared with the experimental data from Jia et al., the incidence of RP was significantly lower than that in the osimertinib group, and there were no deaths in our study. This conclusion undoubtedly provides substantial support for the future use of aumolertinib combined with TRT.

In terms of the risk factors for RP, previous studies have demonstrated that dose parameters such as the total lung V20 and the MLD are risk factors for the occurrence of RP [27]. Considering the above information, our study limited the total lung V20 < 25%, and the median MLD and total lung V20 were 6.36 Gy and 11.5%, respectively. This may also explain why the incidence of RP in our study was lower than that in the osimertinib group of Jia et al. Notably, our study further proved that an ipsilateral lung V20 ≥ 25% was an independent predictive factor of RP. Therefore, controlling dosimetric parameters is a key aspect that needs to be focused on in reducing the incidence of RP. This conclusion may also be helpful for developing more precise treatment plans in the future. The relationship between the tumor volume or GTV and grade ≥ 2 RP has also been demonstrated by previous studies [28], and our study came to the same conclusion, reminding us to be more cautious in delineating the GTV. In addition, Jia et al. reported that a longer overlap time of TRT and TKI was a risk factor for grade ≥ 2 RP [29]. The same trend was also found in our study (OR = 3.68), although the overlap time of TRT and TKI was meaningful according to univariate analysis but meaningless according to multivariate analysis. In addition to the high safety of aumolertinib, this may also be due to the small sample size, which did not significantly affect the results. The specific impact of overlap time on RP needs to be explored in a large sample.

The low incidence of RP may be explained by several therapeutic mechanisms. The first- generation TKI educed G2/M-phase cell cycle arrest and promoted apoptosis through the EGFR signaling pathway, which increased radiation sensitivity while suppressing the proliferation of alveolar epithelial cells and the repair of lung tissue [30, 31]. Researchers found that the action of aumolertinib was optimized by the use of a cyclopropyl group to replace a methyl group on the indole ring of osimertinib, thus allowing potentially greater selectivity against EGFR T790M to mediate the progression and metastasis of lung cancer, thereby reducing damage to lung tissue to some degree [32, 33]. The AENEAS and APOLLO studies also found that the incidences of severe interstitial pneumonitis induced by aumolertinib were only 0.9% and 0%, respectively. Because of the low degree of damage to the pulmonary interstitium, the occurrence of RP was suppressed.

This study has several limitations. First, this was a single-institution, small-sample retrospective study with short follow-up time, thus there were no direct comparisons in efficacy and safety between aumolertinib combined with TRT and osimertinib combined with TRT. Although our study demonstrated good PFS and a tolerable safety of aumolertinib combined with TRT, more studies are needed to further explore difference between aumolertinib an other third-generation TKIs. Second, we controlled the lung dosimetric parameters such as total lung V20 < 25% to reduce the damage of RP. But without analyzing the influencing factors of OS and PFS, we cannot rule out the possibility that limiting lung dose may affect survival. Third, patients who treated with aumolertinib and TRT as second-line therapy were also included in our study. Although previous studies explored the incidence of RP caused by TKI combined with TRT as first-line therapy, there was no study exploring RP of these patients after receiving second-line aumolertinib combined with TRT. In our study, all patients in the second-line group had stopped using the first-generation TKI for a long time before receiving aumolertinib and TRT, and all patients had good lung function without concomitant interstitial pneumonia. Although the impact of first-line therapy on RP cannot be completely ignored, it has been minimized as much as possible.

However, aumolertinib was only recently launched in China, and the number of patients receiving the drug was relatively small, which was also the main reason for the small sample size and short follow-up time in our study. Although third-generation TKI has been considered first-line therapy for EGFR-mutant NSCLC patients, the efficacy and safety of aumolertinib combined with TRT have not been validated. Moreover, the combination of osimertinib and TRT caused a high incidence of pneumonia; thus, further exploration of the impact of aumolertinib combined with TRT on RP is necessary. As the first investigation to demonstrate the superior effect and acceptable safety of aumolertinib combined with TRT in NSCLC patients and to analyze the risk factors for RP, this study has guiding importance for the future clinical application of aumolertinib and provides a reference for further improving the safety of treatment. In addition, patients in this study are currently under continuous follow-up, and the sample size will continue to increase. In the future, we will further explore the influence factors of PFS and OS.

## Conclusion

In summary, concurrent aumolertinib and TRT achieved good local control and a tolerable incidence of RP. The risk factors for grade ≥ 2 RP in NSCLC patients are the GTV and ipsilateral lung V20. Therefore, limiting dosimetric parameters may be helpful for reducing the incidence of RP. This work provides preliminary data on the guidance for concurrent aumolertinib and TRT in clinical practice. In the future, it will be important to carefully control the radiation dose while considering the patient’s physical condition and the size of the primary lesion to achieve better treatment effects.

## Data Availability

All the data generated or analyzed during this study are included in this published article. The datasets used and/or analyzed during the current study are available from the corresponding author.

## Abbreviations

EGFR-TKIs: Epidermal growth factor receptor tyrosine kinase inhibitors
NSCLC: Non-small cell lung cancer
RP: Radiation pneumonitis
TRT: Thoracic radiotherapy
CCRT: Concurrent chemoradiotherapy
CT: Computed tomography
GTV: Gross tumor volume
CTV: Clinical target volume
PTV: Planning target volume
DVH: Dose and volume histogram
AUC: Area under the curve
ROC: Receiver operating characteristic
OR: Odds ratio
CI: Confidence interval
PD: Progressive disease
SD: Stable disease
MLD: Mean lung dose
ORR: Objective response rate
